# Design and Synthesis of Simplified Polyketide Analogs: New Modalities beyond the Rule of 5

**DOI:** 10.1002/cmdc.202100150

**Published:** 2021-05-05

**Authors:** Dirk Menche

**Affiliations:** ^1^ Kekulé-Institut für Organische Chemie und Biochemie Universität Bonn Gerhard-Domagk-Strasse 1 53121 Bonn Germany

**Keywords:** Natural products, polyketides, rule of 5, new pharmaceutical modalities, non-covalent networks

## Abstract

Natural products provide important lead structures for development of pharmaceutical agents or present attractive tools for medicinal chemistry. However, structurally complex and thus less accessible metabolites defying conventional drug‐like properties, as expressed by Pfizer's rule of five, have received less attention as medicinal leads. Traditionally, research focus has been on realizing total syntheses rather than developing more readily available analogs to resolve the critical supply issue. However, very recent studies with complex myxobacterial polyketides have demonstrated that considerable structural simplification may be realized with retention of biological potencies. The context, underlying rationale and importance of tailored synthetic strategies of three such case studies are presented, which may inspire further related activities and may eventually help exploiting the largely untapped biological potential of complex metabolites in general.

## Graphical abstract

**Natural product simplifications**: Recent studies with complex myxobacterial polyketides demonstrate that their architectures may be considerably simplified with retention of biological potencies, suggesting that such studies may be generally attractive in helping to advance the largely untapped biological potential of elaborate complex metabolites.

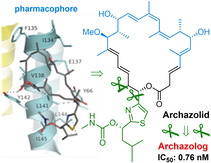

## Introduction

The exquisite and varied architectures of natural products continue to provide a rich pallet for discovery in medicinal chemistry. Whether they are used to probe biological mechanisms or provide the basis for pharmaceutical drug discovery, serve as lead structures for development of novel therapeutic agents, natural metabolites continue to command attention. In fact, it has been estimated that more than 60 % of new chemical entities introduced as drugs during the last two decades are, or were inspired by, natural products.^[1]^ However, structurally complex and consequently less accessible metabolites have received much less attention as pharmaceutical leads.^[1]^ Importantly, they defy common drug‐like properties as expressed by Lipinski's rule of five, such as overall size and number of polar group.^[2]^ Also, supply issues are often not resolved. Traditionally, providing these scarce metabolites has been addressed by total synthesis, which despite impressive progress still often generates only small amounts of the target compounds.

**Figure 1:**
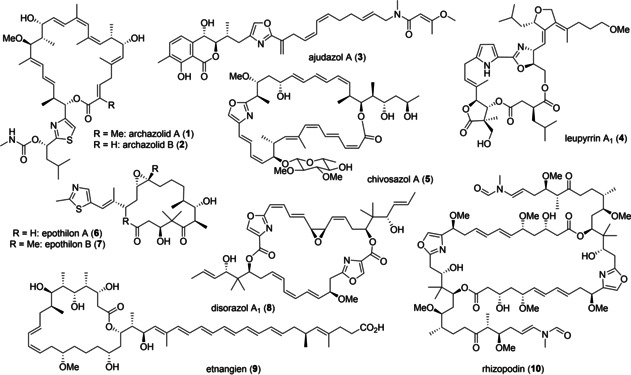
Complex polyketides from myxobactria: a largely untapped pool of new modalities for medicinal and pharmaceutical chemistry.

From the perspective of medicinal chemistry, complex polyketides are particularly attractive study objectives, as they may be characterized by a high degree of structural diversity and stereochemical complexity in combination with a broad range of important biological properties.^[3]^ Myxobacteria present a very rich source of structurally novel and biologically intriguing polyketides.[^3a^, ^3b^] It has been estimated, that more than 60 novel architectures have been discovered from these soil‐living bacteria. As shown in Figure 1, prominent examples include the V‐ATPase inhibitory archazolids (**1**, **2**),^[4]^ mitochondrial NADH‐dehydrogenase binding ajudazols (**3**),^[5]^ or antifungal leupyrrins (**4**).^[6]^ The chivosazols (**5**),^[7]^ epothilones (**6**, **7**),^[8]^ disorazoles (**8**)^[9]^ and rhizopodin (**10**)^[10]^ in turn interact specifically with the cell cytoskeleton binding to F‐ or G‐actin (**5**, **10**) or tubulin (**6**, **8**), while antibacterial etnangien (**9**)^[11]^ targets RNA‐polymerase. Notably, in all cases these polyketides belong to the most potent inhibitors known for the respective molecular targets. However, despite these highly attractive profiles, only the epothilones have been further developed as anticancer agents.^[10]^

While all these compounds have been obtained by impressive total syntheses,^[12]^ the many steps required to access these elaborate structures have usually impeded access to more than mg amounts. Conversely, more accessible, simplified analogues with similar or even improved activities or pharmacokinetic properties are much more desirable, as these may eventually resolve the critical supply issue. However, only few such studies have been reported.[^2e^, ^13^] Also, the authentic metabolites are widely considered as evolutionary optimized, and analog studies indeed often result in considerable loss in activity.[^13^, ^14^] However, recent findings suggest that such general scepticism may not be justified and remarkable analogs have been found that retain potent bioactivities, despite considerable simplifications.[^15^, ^16^, ^17^, ^18^] Herein, we discuss three case studies focusing on the archazolids (**1**, **2**),^[16]^ ajudazols (**3**)^[17]^ and leupyrrins (**4**)^[18]^ where rational approaches in combination with tailored synthetic strategies have led to the discovery of truncated and more readily available analogs, that retain potent biological properties of the parent natural products and may eventually help advancing the biological profile of these complex polyketides as new pharmaceutical modalities beyond the classical rule of five.

## From Archazolids to Archazologs

The first example discussed, the archazolids A and B (**1**, **2**, Figure 1) present extremely potent antiproliferative agents that inhibit the range of various cancer cell lines in low nano‐ or even sub‐nanomolar concentrations.[^4a^, ^19^] With a molecular wight of 739 (**1**), the certainly exceed the size as well as complexity usually observed for pharmaceuticals. On a molecular level, they demonstrate highly potent inhibitory activities against vacuolar‐type ATPases (=V‐ATPases), key regulatory enzymes of broad range of biochemical processes.^[20]^ For a long time, these proton pumps have not been considered as drugable targets, due to inherent selectivity issues of these omnipresent multimeric protein complexes.^[20]^ However, in recent years the pioneering work of the Vollmar group and others, has shown that these macrolides may indeed exert very potent and surprisingly selective antitumoral activities in a number of relevant models as well as in *in vivo* studies.^[21]^ This has rendered these polyketides emerging new lead structures for development of novel types of antitumoral agents. However, their further development has been severely hampered by supply issues which could not be resolved by total syntheses mainly due to the many steps required to access their unique polyunsaturated macrolactones, including eight stereogenic centers.[^12a^, ^22^] Originally, also only a few SAR data have been obtained, relying mainly on natural product derivatizations,^[23]^ novel natural derivatives^[24]^ or drastically simplified fragments with low bioactivity.^[25]^

**Scheme 1:**
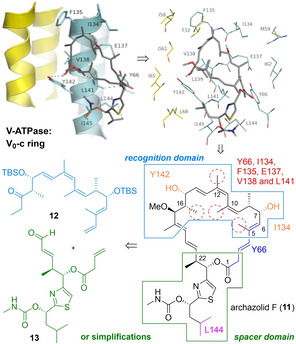
Non‐covalent interaction network of the archazolids with the V_o_ ring of V‐ATPases: design of a tailored synthetic strategy for archazolids and analogs.

Our analog studies were initiated by developing a molecular model for the non‐covalent archazolid V‐ATPase interactions. In detail, the binding site at the membrane bound V_0_ subunit c was determined by displacement and crosslinking studies.^[26]^ Based on detailed mutagenesis studies,^[26a]^ in combination with molecular modeling, EPR measurements,^[26b]^ and first SAR data,[^23^, ^24^] a framework for the interaction network could then be proposed.^[27]^ In detail, key interactions are observed for the Northern part with residues Y66, I134, F135, E137, V138, and L141, while hydrophobic interactions are much less pronounced to the Southern area (L144) (Scheme 1, top part). Therefore, it was rationalized that the Northern part would be critical for target interaction, presumably as recognition domain, while more flexibility was suggested for the Southern region. Based on this analysis, a tailored synthetic strategy was devised, involving a separate preparation of the Northern pharmacophore **12** and a modular attachment of Southern subunits **13**.^[28]^ Along these lines, a first total synthesis archazolid F (**11**), the most potent and least abundant archazolid,^[24c]^ was also planned.

**Scheme 2:**
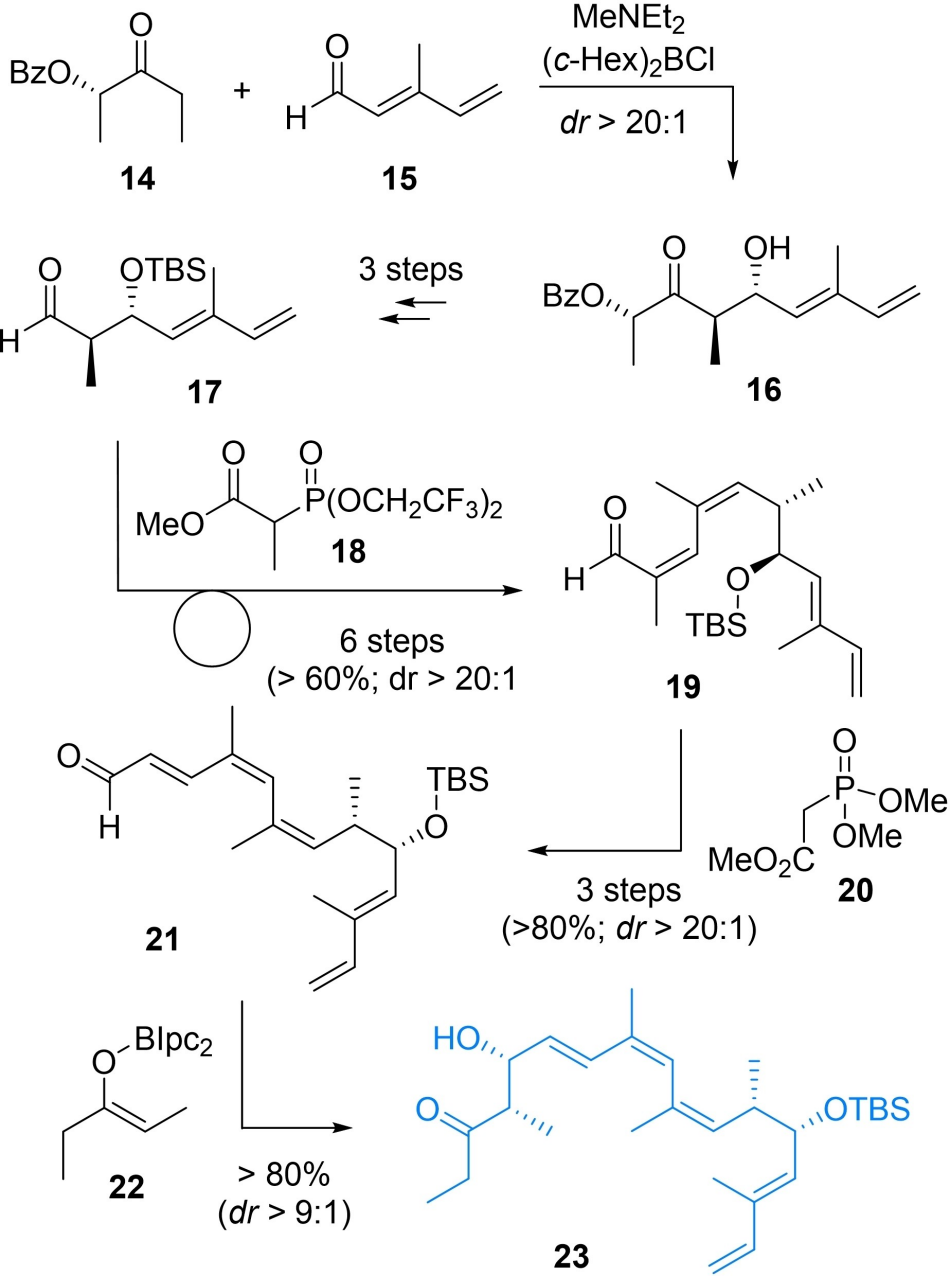
Synthetic and reagent design for a high‐yielding and robust synthesis of the northern archazolid pharmacophore.

As shown in Scheme 2, synthesis of Northern fragment **23** serves as an instructive example of how suitable synthetic design and careful reagent choice allow access to even elaborate polyketide segments in a robust and scalable manner. In detail, a sequence of reliable aldol and olefination methodology enabled access to Northern pharmacophore **23** in gram quantities with an impressive overall yield (27 % for R=CH=CH_2_). The sequence involved elaboration of readily available HWE products of type **15** by a Paterson *anti*‐aldol reaction with chiral auxiliary **14**, proceeding in very good yield and excellent selectivity in all cases. After facile standard manipulations, two consecutive Still‐Gennari reactions with phosphonate **18** and a subsequent HWE reaction using **20** of derived **19** then set the characteristic (*Z*,*Z*,*E*)‐trieneoate **21**, likewise in excellent yield and selectivity, before a final Ipc‐mediated aldol coupling generated the required *syn*‐pattern in an efficient manner. Notably, this sequence was readily applicable to a several analogs, differing in the substitution pattern R, in high yield, demonstrating the generality and reliability of such sequences for elaborate polyketide fragment synthesis.

**Scheme 3:**
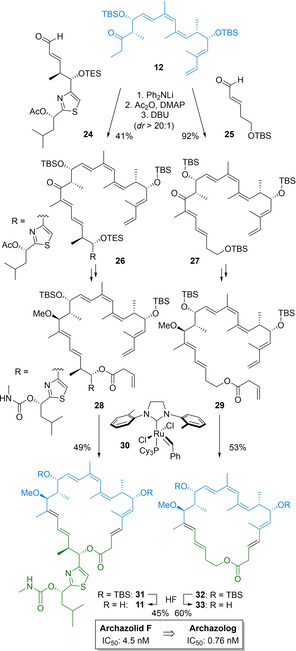
Diversity oriented synthesis of archazolids and archazologs by a late‐stage diversification strategy lead to the discovery of a highly potent simplified analog **33** (IC_50_ values: growth inhibition of 1321N1 astrocytoma cells).

A diversity oriented approach was then implemented for a modular synthesis of archazolids and archazologs. This tailored synthetic strategy allowed rapid access both to the parent natural product as well as to carefully designed analogs in a versatile manner.^[16]^ As shown in Scheme 3, this involved development and implementation of an adventurous aldol condensation of Northern subunit **12** with various aldehydes, such as authentic **24** or surrogate **25** and a challenging RCM reaction of an octane precursor (**28** or **29**). Based on suitable reagent and catalyst design, either conversion proceeded with good to excellent selectivity and yield, considering the high synthetic challenge posed on these key transformations, in particular for ring closure, comparing favorably to previous approaches.^[12a]^ In combination with conventional functional group manipulations of enoates **28** and **29**, and a final deprotection of **31** and **32**, a first total synthesis of archazolid F (**11**) and a range of archazologs, including **33**, could then be realized.

Importantly, simplified archazolog **33** proved to be highly active, with activities in the low nano‐ or sub‐nanomolar range that even excelled those of archazolid F.^[16a]^ These data demonstrate that the complex archazolid structure may be dramatically simplified without loss of potency, thus also confirming our pharmacophore hypothesis. It also shows that a reliable model for target binding may be proposed, even for complex interaction networks of elaborate polyketides with a transmembrane target, based on state of the art biochemical methods and advanced computational techniques. Many other archazologs can now be envisioned, which are much more readily accessible, potentially resolving the supply issue and enabling the further development of this promising novel class of potent anticancer drugs.

## From Ajudazol A to Ajudazol T

Inspired by this remarkable discovery, we turned our attention to a second class of natural products, the ajudazols A (**3**, MW: 590) and B (**34**, MW: 992, Scheme 4), structurally unique polyketides,^[5]^ that are characterized by a rare isochromanone with two vicinal *anti*‐configured hydroxyls and an extended side chain with an oxazole, a *Z,Z*‐diene and a terminal methoxybutenoic acid methylamide as characteristic features. Being the most potent known inhibitors of mitochondrial complex I NADH‐dehydrogenase known to date,[^5a^, ^5b^] they are likewise characterized by a highly attractive biological profile. However, further exploration has only been hampered by supply issues but also by facile degradation of the sensitive isochromanone moiety.

**Scheme 4:**
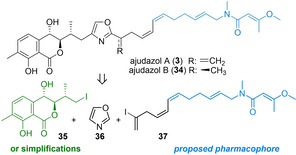
Innovative oxazole diversification strategy for a modular synthesis of ajudazols and selected analogs.

Based on this observation, an innovative synthetic strategy was designed for a modular replacement of this sensitive heterocycle.^[17]^ As shown in Scheme 4 this relies on a modular oxazole diversification approach. This allows for a more facile applicability to carefully designed analogs, also as compared to a more conventional cyclodehydration strategy, commonly used for elaborate oxazole natural products, including a total synthesis of ajudazol B (**34**).[^5c^, ^29^] Inspired by structural analogies to related natural products in combination with target‐inhibitor x‐ray studies,^[14b]^ it was envisioned that the terminal unsaturated amide motif would be part of the pharmacophore and consequently, this subunit was retained.

**Scheme 5:**
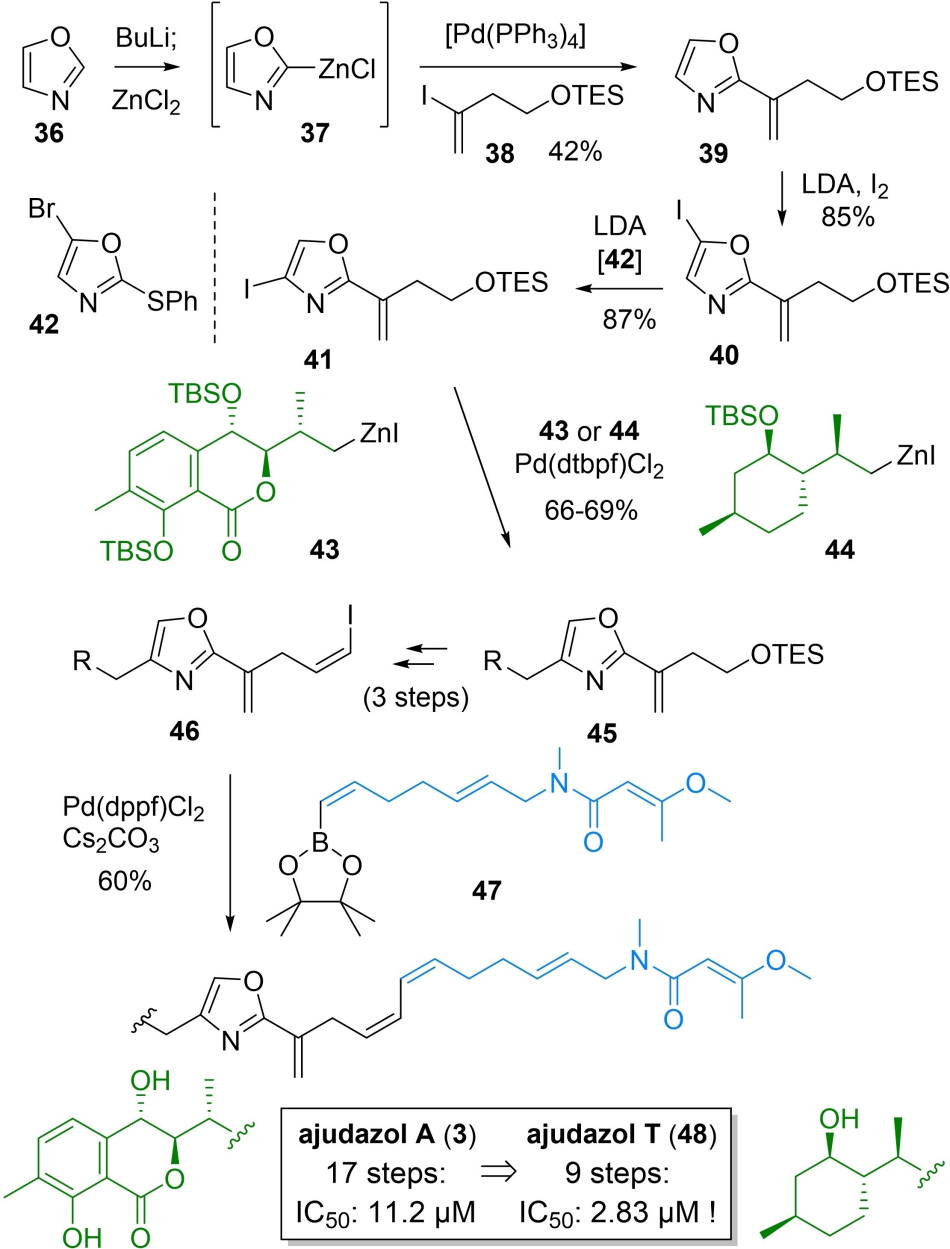
Diversity oriented approach for a joint first total synthesis of ajudazol A and more readily available, but still potent ajudazol T (IC_50_ values against: T cell leukemia cells).

As shown in Scheme 5, implementation of our oxazole diversification strategy was initiated by selective lithiation of unsubstituted oxazole **36**, transmetalation with ZnCl_2_ and an efficient Negishi cross coupling of resulting organyle **37** with vinyl iodide **38**, using specifically developed protocols.^[17]^ Subsequent C^5^‐iodination and an innovative halogen dance reaction of derived **40** towards 4‐iodooxazole **41** proceeded smoothly following a procedure likewise advanced in our group, involving catalytic amounts of **42** (74 %, 2 steps). Presumably, this mechanistically intriguing reaction is initiated by a deprotonation of **42** at C4, followed by a first lithium halide exchange with **40** giving C5 lithiated analog of **40** and 4‐iodo‐derivative of **42**. These two intermediates then undergo a second lithium halide exchange with regeneration of catalyst **42** and formation of C5‐lithiated **41** which hydrolyzes upon workup to the desired product.^[30]^ Negishi cross coupling with simplified Western heterocycle **44** then proceeded in high yield (69 %), demonstrating the usefulness of our approach for selective oxazole derivatization.^[17]^ Besides its structural similarity to authentic **43**, simplified was also selected due to its ready availability in two steps from commercial isopulegol.^[17]^ The derived terminal vinyl iodide **46**, was then converted to desired analog **48** by a Suzuki coupling with Eastern fragment **47** using Pd(dppf)Cl_2_ in presence of Cs_2_CO_3_ and deprotection with buffered HF⋅pyridine. Synthesis of boronate **47** involved either a Wittig reaction or a cross metathesis for the central *E*‐olefin in combination with a Rh‐catalyzed trans‐hydroboration.[^5c^, ^29^] Following this approach, a first total synthesis of ajudazol A (**3**) was realized together with simplified ajudazol T (**48**), by modular attachment of either the original isochromanone **43** or simplified terpene **44**. Importantly, first biological data revealed that simplified ajudazol T (**48**) retained biological activity of the parent compound ajudazol A (**3**) against T cell leukemia cells, confirming our hypothesis of the terminal Eastern butenamide motif to be part of the pharmacophore. However, in contrast to the parent ajudazol A (**3**) it is much more readily available (9 vs 17 steps for **3** or 24 steps for **34**) as well as much more stable, demonstrating again that complex polyketide structure may be simplified based on a suitable analog design and a tailored synthetic strategy, which highlights the potential of commercially available oxazole **36** as a direct starting material for complex oxazole containing natural products.

## From Leupyrrins to Leupylogs

Finally, the third polyketide class discussed within this overview are the leupyrrins. They display highly potent antifungal activities in nanomolar concentrations,^[6]^ together with moderate antiproliferative and anti‐HIV activities.^[31]^ While they efficiently inhibit DNA, RNA, and protein syntheses, conventional molecular targets are not addressed,^[6]^ which may attribute to an unusual molecular interaction site. As exemplified by the parent metabolite leupyrrine A_1_ (**4**, MW: 738, Scheme 6), their unique architectures are characterized by an 18‐membered nonsymmetric macrodiolide core incorporating an unusually substituted γ‐butyrolactone, a pyrrole and an oxazoline heterocycle ring in combination with a side chain containing a unique dihydrofuran with two exocyclic alkylidens.

**Scheme 6:**
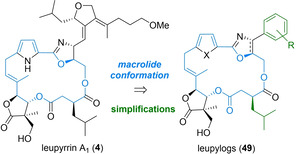
SAR design of simplified leupylog, addressing unfavourable polyene shifts in the furan side chain.

As before, our synthetic design was based on a modular total synthesis of the authentic natural products in combination with selected analogs.^[18]^ A particular focus was placed on simplification and stabilization of the sensitive dihydrofuran segment, being prone to olefinic rearrangements. Based on a certain biological flexibility of the natural side chain, it was envisioned that the alkyliden units may be stabilized by incorporation into an aromatic moiety, leading to stabilized as well as simplified leupylogs of type **49**.^[18]^ Also, a first evaluation of natural product derivatives suggested, that the macrocyclic ring would have to be retained for biological potency.^[18]^

**Scheme 7:**
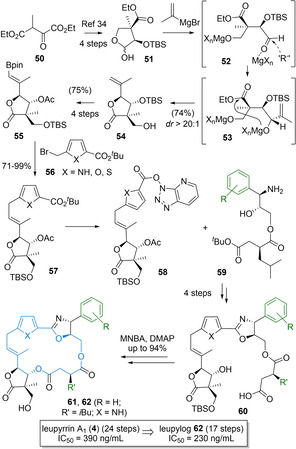
Novel tandem methodology enable a rapid synthesis of leupylogs, leading to the discovery of a highly potent but much more readily available analog, that retains the powerful antifungal activity of the parent natural product (IC_50_ values against: *Rhodotorula glutinis*).

Within this context, a particular emphasis was placed on novel domino reactions, generally accepted as a key enabling technology for rapid access to elaborate polyketide functionalities.^[32]^ Within this context, an innovative one‐pot process for synthesis of the densely functionalized butyrolactone segment was developed,[^6b^, ^12h^] which proved to be by far superior to alternative approaches.^[31]^ As shown in Scheme 7 (top part), this involved opening of readily available lactol **51**,^[34]^ nucleophilic addition of *iso*‐propenylmagnesium bromide to the corresponding aldehyde **52**, followed by an intramolecular trans‐esterification to **54**. This process proceeded in high yield and excellent selectivity.^[6b]^

After further decoration, involving a beneficial cross metathesis with a carefully selected pinacol ester, derived boronate **55** was elaborated by an adventurous sp^2^‐sp^3^‐coupling strategy. Following this unusual junction, various heterocyclic building blocks could be attached allowing for high modularity, relying on suitable protocol development for such less common Suzuki‐reaction at such benzylic positions. A condensation strategy of derived activated ester **58** ^[35]^ with amino alcohol **59** to access oxazoline **60** proved optimal and also allowed facile access to saturated analogs (not shown). Finally, Shiina macrolactonizations proceeded in high yield for a range of leupyrrins and leupylogs (**61**).^[18]^ In total, a general SAR studied with a set of around 15 leupyrrins and leupylogs of synthetic and natural origin was realized revealing important SAR insights for range of structural subunits.^[18]^ Ultimately, this lead to the discovery of considerably simplified leupylogs that retained the highly potent antifungal activity of the parent natural products but incorporates a stable and readily available aromatic side chain. A concise strategy for the synthesis of leupylogs could be realized, requiring 17 steps for **62** in the longest linear sequence as compared to 24 steps for leupyrrin A (**4**).

## Conclusions and Outlook

In summary, these three case studies have demonstrated that simplified series of analogs of complex polyketides may be developed that retain the powerful potency of the parent natural products. These examples show that the overall structure can be considerably simplified, which is quite remarkable as only few such examples have been described in the context of complex natural macrolides and polyketides.[^2e^, ^13^] Also quite remarkable activities have been observed for simplified derivatives of the chivosazoles and spirangien by the group of Kalesse,[^15a^, ^15b^] and the disorazoles by the groups of Kalesse^[15c]^ and Nicolaou.^[15d]^ Along these lines, organic synthesis serves as a key technology in providing tailored synthetic strategies enabling a versatile access to carefully designed analogs that could otherwise not be obtained. Novel tandem reactions for a rapid access to key structural features as well as novel protocols for unusual bond junctions enable useful and as well as more rapid access to even elaborate functional compounds. Also, these studies demonstrate that even large scale approaches may be realized through multi‐step sequences, depending on suitable reagent and route design. These examples also demonstrate the importance of a suitable concept for analog development. Along these lines, advanced biochemical techniques and molecular modeling proved very helpful for an educated selection of structural simplification and/or the design of improved derivatives. It is expected, that continuous advances in *in silico* methods will have a profound impact on such studies, in particular in modelling the highly complex underlying non‐covalent interaction networks that are critical both for conformational control as well as target inhibitor correlations. It will be interesting to follow the further fate of these polyketides as well as complex metabolites in general. Eventually, such analog may be generally attractive in advancing the biological potential of complex natural products as a largely untapped pool of new modalities in pharmaceutical and medicinal chemistry that do not adhere the classical rule of five.

## Conflict of interest

The author declares no conflict of interest.
